# Short physical performance battery is not associated with falls and injurious falls in older persons: longitudinal data of the SCOPE project

**DOI:** 10.1007/s41999-024-00941-y

**Published:** 2024-02-28

**Authors:** Ellen Freiberger, Paolo Fabbietti, Andrea Corsonello, Fabrizia Lattanzio, Cornel Sieber, Lisanne Tap, Francesco Mattace-Raso, Johan Ärnlöv, Axel C. Carlsson, Regina Roller-Wirnsberger, Gerhard Wirnsberger, Rafael Moreno-Gonzalez, Francesc Formiga, Sara Lainez Martinez, Pedro Gil, Tomasz Kostka, Agnieszka Guligowska, Ilan Yehoshua, Itshak Melzer, Robert Kob, the SCOPE investigators

**Affiliations:** 1 Department of Internal Medicine-Geriatrics, Institute for Biomedicine of Aging (IBA), Friedrich-Alexander-Universität Erlangen-Nürnberg, Erlangen, Germany; 2 Center for Biostatistic and Applied Geriatric Clinical Epidemiology, Italian National Research Center on Ageing (IRCCS INRCA), Ancona and Cosenza, Via S. Margherita 5, 60121 Ancona, Italy; 3 Department of Pharmacy, Health and Nutritional Sciences, University of Calabria, Rende, Italy; 4 Scientific Direction, Italian National Research Center on Aging (IRCCS INRCA), Ancona, Italy; 5 Section of Geriatric Medicine, Department of Internal Medicine, Erasmus University Medical Center Rotterdam, Rotterdam, The Netherlands; 6 Division of Family Medicine and Primary Care, NVS Department, Karolinska Institutet, Alfred Nobels Allé 23, 141 83 Huddinge, Sweden; 7 Academic Primary Health Care Centre, Region Stockholm, Stockholm, Sweden; 8 School of Health and Social Studies, Dalarna University, Falun, Sweden; 9 Department of Internal Medicine-Geriatrics, Medical University of Graz, Graz, Austria; 10 Department of Internal Medicine, Medical University of Graz, Graz, Austria; 11 Geriatric Unit, Internal Medicine Department, Bellvitge University Hospital-IDIBELL-L’Hospitalet de Llobregat, Barcelona, Spain; 12 Geriatric Department, Hospital Clínico San Carlos, Martín Lagos S/N, 28040 Madrid, Spain; 13 Department of Geriatrics, Healthy Ageing Research Centre, Medical University of Lodz, Lodz, Poland; 14 Department of Physical Therapy, Recanati School for Community Health Professions at the Faculty of Health Sciences, Ben-Gurion University of the Negev, Beersheba, Israel; 15 Maccabi Healthcare Services, Tel Aviv, Israel

**Keywords:** Short physical performance battery, Falls, Injurious falls, Physical function, Longitudinal study

## Abstract

**Introduction:**

Falls and fall-related injuries in older persons are a major public health problem. Our objective was to study the predictive value of the Short Physical Performance Battery (SPPB) in the cohort of the SCOPE project on falls, injurious falls, and possible difference of prediction between indoors and outdoors falls.

**Methods:**

For this sub-study of the SCOPE project participants reporting no falls at baseline, and survey data on falls at the 12-month and 24-month follow-up were included. Participant´s characteristics were assessed during the baseline interview and medical examinations. Falls as well as injurious falls and fall circumstances were obtained self-reported. SPPB and its association with fallers vs. no fallers at 12 and at 24 months were studied with logistic regression models.

**Results:**

The 1198 participants had a median age of 79 years (77–82), and a median SPPB of 10 (8–11), with a 52.5% of female. A total of 227 and 277 falls (12- and 24- month visits, respectively) were reported. In the crude model, the SPPB sum scores (*p* < 0.001) as well as most single item scores were significant different between fallers and non-fallers over time. However, the association was attenuated in models adjusted for age, sex, marital status, number of medications, quality of life, handgrip strength, and muscle mass [e.g., 12 months; OR 0.94 (0.87–1.02)]. While SPPB fails to differentiate between injurious and non-injurious falls (*p* = 0.48), a lower SPPB score was associated with falls at home (*p* < 0.01) after 24 months.

**Conclusion:**

SBPP was not able to significantly predict the risk of falling as well as experiencing an injurious fall.

**Trial registration:**

This study was registered prospectively on 25th February 2016 at clinicaltrials.gov (NCT02691546).

## Key summary points

**Aim:**

Our objective was to study the predictive value of the Short Physical Performance Battery (SPPB) in the cohort of the SCOPE project on falls, injurious falls, and possible difference of prediction between indoors and outdoors falls.

**Findings:**

No association of SPPB and falls was found in models adjusted for age, sex, marital status, number of medications, quality of life, handgrip strength, and muscle mass. While SPPB fails to differentiate between injurious and non-injurious falls (*p* = 0.48), a lower SPPB score was associated with falls at home (*p* < 0.01) after 24 months.

**Message:**

SBPP was not able to significantly predict the risk of falling as well as experiencing an injurious fall.

## Introduction

Falls and fall-related injuries in older persons are a major public health problem with about 35–40% of older persons over 65 years falling per year and about 1 in 40 of these fallers will be hospitalized [4]. Population-based research demonstrated that 10% of older fallers experience at least two falls per year [5]. In about 10% of all falls, serious injuries, such as fractures, joint dislocation sprains, or concussions, occur and lead to emergency visits or hospitalization. Therefore, falls are a high burden to the public health care costs as the Global Burden of Disease, Injuries and Risk Factor Study estimated that the burden of falls were ranked as the eighteenth leading cause of age-standardized rates of disability-adjusted life years in 2017 [3, 6]. In older persons with and without falls’ experience, concerns about falling play an important role in activity restriction and reduced quality of life and thus fueling a negative circle of deconditioning, weakness, and possible gait impairments [4], and finally will end in more falls. As the number of falls is estimated to increase worldwide in the future [3], identifying older persons at risk of falls is of utmost interest and is emphasized by most international and national guidelines [3, 7, 8].

A recent umbrella review, being part of the World Falls Guidelines (WFG) group, found a vast heterogeneity of different assessment tools in the area of physical function including gait, balance, and functional mobility [9]. The tools included TUG, Berg Balance test, gait speed tests, dual task assessments, single leg stance, and functional reach [9]. Due to the lack of reviews on the Short Physical Performance Battery (SPPB), the SPPB could not be included. Albeit, SPPB is a clinical testing procedure easily to manage, needs little equipment, and space. Furthermore, the SPPB is one of the most validated, standardized, and established geriatric assessment measuring lower limb functional muscle strength (five-repetition sit-to-stand chair test, 5STS), and balance and gait speed [10, 11]; it addresses three of the most cited physical risk factors on falls. As balance, gait deficits as well as lower limb weakness are the most important risk factors for falls the SPPB including all three dimensions seems to be an excellent assessment tool to identify older persons at fall risk [4].

To extend current knowledge on the SPPB predicting falls and injurious falls, we made use of the SCOPE study providing us with data on falls, injurious falls, as well as information on location of falls—indoors vs. outdoors falls. Our first objective was to study the predictive value of the SPPB in the cohort of the SCOPE project on falls. Second, the prediction of experiencing an injurious fall by SPPB was analyzed. Furthermore, the ability of the SPPB to divine if falls will occur indoors and outdoors was the third objective.

## Methods

The SCOPE study is an observational, multinational, multicenter, prospective cohort study targeting chronic kidney disease screening in community-dwelling older persons throughout Europe. Its study design has been reported in depths elsewhere [12]. Briefly, SCOPE is a multicenter 2-year prospective cohort study involving patients older than 75 years attending outpatient services in participating institutions in Austria, Germany, Israel, Italy, The Netherlands, Poland, and Spain. Only few exclusion criteria were applied, e.g., life expectance less than 6 months, severe cognitive impairments, or end-stage renal kidney disease. Participants were requested to sign a written informed consent before entering the study. The study protocol was approved by ethics committees at all participating institutions and complies with the Declaration of Helsinki and Good Clinical Practice Guidelines. Participants were re-assessed after 12 and 24 months.

For this sub-study on SPPB predicting falls, only participants who reported no fall within the last 12 months at baseline were included. Therefore, our sample consisted of 1198 participants of the full sample of the 2461 SCOPE participants initially enrolled in the study.

### Study design

Covariates for this sub-study were selected from the SCOPE data pool to match different domains of fall-risk factors, and related to fall-risk screening items from the American Geriatric Society (AGS), the older fall prevention Initiative of the CDC (STEADI) initiative [13, 14]. We only excluded the variable fear of falling, as we had published results on this topic with the SCOPE data already [15].

### Sociodemographic, anthropometry

Participants’ characteristics were assessed during the baseline interview and medical examination. Demographic variables included age, sex, educational level, and self-reported living-status (living alone vs. living with others). Body mass index (BMI) was calculated by body weight and height and expressed as kg/m^2^. Muscle mass was measured in lying condition using bioelectrical impedance analysis (BIA; AKERN BIA 101 New Edition 50 kHz monofrequency device, AKERN SRL, Florence, Italy).

### Questionnaires on comprehensive geriatric assessment, multimorbidity and self-reported history of falls, concerns about falling, and quality of life (QoL)

The comprehensive geriatric assessment (CGA) was performed including Mini-Mental State Examination (MMSE)/cognitive status [16], 15-item Geriatric Depression Scale (GDS)/mood [17], Basic (ADL), and Instrumental Activities of Daily Living (IADL)/self-reported disability [18, 19].

Diseases were documented and analyzed using Cumulative Illness Rating Scale for Geriatrics (CIRS-G Total Score)/overall comorbidity [20] and number of prescribed medications were assessed.

Health-related quality of life was rated by Euro-Qol 5D [21] questionnaire, including total score and QoL visual analogue scale (EQ-VAS). Participants were asked to evaluate their overall health today on a vertical visual analogue scale, ranging from 0 “worst possible” to 100 “best possible”.

Urinary incontinence was defined as at least one moderate or big problem in dripping or leaking urine, weak urine stream or incomplete emptying, waking up to urinate, and need to urinate frequently during the day. Stages of urinary incontinence were obtained with the lower urinary tract symptoms (LUTS) questionnaire.

### Muscle strength and physical performance measures

Maximum handgrip strength of three alternating attempts for each hand was assessed with a hydraulic dynamometer (Model J00105 JAMAR Hydraulic Hand, Lafayette Instrument Company, USA). The SPPB included timed gait speed, five chair-stand test (time to rise from a chair and return to the seated position 5 times without using arms), and hierarchical balance test (ability to stand with the feet together in the side-by-side, semi-tandem, and tandem positions). The SPPB assess these domains with established cutoffs and group these scores into categories ranging from 0 (worst performance) to 4 points (best performance). The maximum total score in the SPPB is 12 points. The SPPB assessment followed the protocol by Guralnik et al. 2000 [22].

### Falls

Falls were self-reported obtained with the question “How many times have you fallen in the past 12-months?” at all three timepoints. A fall was defined as any fall caused by accident, environment, collapse, dizziness, balance/gait impairments including caused by trips, stumbling, quick movements or lost balance, or other reasons. Further information addressing injuries or location of a fall were also obtained. Location was asked as “Inside the home”, “inside a building but not at home”, or “outside”. Inside a building but not at home and outside were grouped for the analysis as “No-Home” falls, whereas inside the home were grouped as “Home” falls. Injuries were obtained by the categories of fractures, treated injury, untreated injury, and no injury. The first three options were grouped as “injurious falls” and the latter as “non-injurious falls”.

### Statistical analysis

First, descriptive analysis of the study population grouped according to fallers and non-fallers categories was provided at 12- and at 24-month follow-ups. All continuous variables were not normally distributed; therefore, they were expressed by median (interquartile range). Categorical data were reported as number (percentage). Chi-square test was used to compare categorical variables, while Mann–Whitney non-parametric test was used for continuous ones.

Then, SPPB comparisons between fallers and non-fallers, injurious falls and non-injurious falls, home fallers, and no-home fallers, respectively, were performed at 12-month and 24-month follow-ups.

To study SPPB and its association with fallers vs. no fallers at 12 and at 24 months, logistic regression models were built. Crude, adjusted models for age and sex, and for other significant variables, were created.

Statistical analysis was carried out using SPSS for Win V24.0 (SPSS Inc., Chicago, IL, USA). A *p* value < 0.05 was considered statistically significant.

## Results

**Fig. 1:**
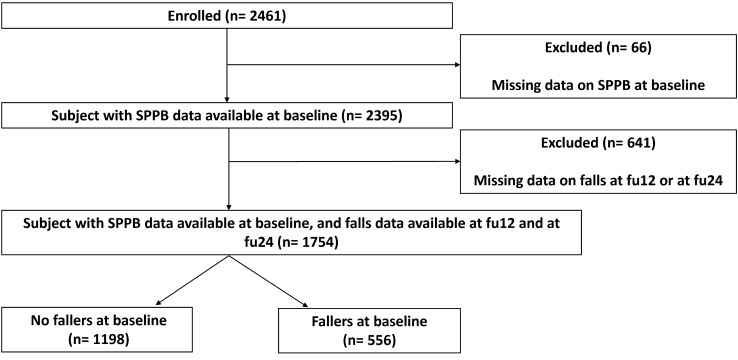
Flowchart of the selection of participants for this sub-study

For this sub-study of the SCOPE project, 1198 participants reporting no falls at baseline were included. The median age of the included SCOPE participants were 79 years (77–82) with a 52.5% of female), and a median SPPB of 10 (8–11). At the 12-month follow-up, 227 reported at least one fall, whereas 991 participants did not experience any fall. At the 24-month follow-up of the 1198 participants, 277 reported at least one fall and 921 did not experience a fall. A flowchart of the selection of participants for this sub-study is depicted in Fig. 1.

Table 1 displays the baseline characteristics of fallers at 12- and 24-month follow-up vs. non-fallers.

**Table 1:** Baseline characteristics comparing fallers and non-fallers at 12-month and 24-month follow-up

Variable	Fallers at follow-up 12 months *N* = 227	Non-fallers at follow-up 12 months *N* = 971	*P*	Fallers at follow-up 24 months *N* = 277	Non-fallers at follow-up 24 months *N* = 921	*P*
Age (years)	79.0 (77.0–83.0)	79.0 (77.0–82.0)	**0.009**	80.0 (77.0–83.0)	79.0 (77.0–82.0)	**< 0.001**
Sex (female), *n* (%)	135 (59.5)	494 (50.9)	**0.020**	169 (61.0)	460 (49.9)	**0.001**
Mini-Mental Score Examination (MMSE)	28.0 (26.3–29.0)	28.0 (27.0–29.0)	0.095	28.0 (26.3–29.0)	28.0 (27.0–29.0)	0.474
Educational level (years)	11.0 (8.0–15.0)	12.0 (8.0–15.0)	0.952	12.0 (8.0–15.0)	12.0 (8.0–15.0)	0.975
Marital status, *n* (%)			**0.046**			**< 0.001**
Single	14 (6.2)	45 (4.6)		22 (7.9)	37 (4.0)	
Married/living with a partner	120 (52.9)	611 (62.9)		140 (50.5)	591 (64.2)	
Separated/divorced	12 (5.3)	45 (4.6)		16 (5.8)	41 (4.5)	
Widowed	81 (35.7)	270 (27.8)		99 (35.7)	252 (27.4)	
Height (cm)	161.0 (155.0–169.0)	163.0 (156.0–170.0)	0.067	161.0 (154.0–168.0)	163.0 (156.0–170.0)	**0.003**
Weight (kg)	72.0 (63.5–81.0)	73.6 (64.0–82.6)	0.337	72.5 (63.1–81.2)	73.3 (64.0–82.2)	0.539
BMI (kg/m^2^)	27.4 (24.8–29.9)	27.2 (24.8–30.2)	0.999	27.7 (25.1–30.5)	27.0 (24.7–30.0)	0.082
Instrumental Activities of Daily Living Score	2.0 (0.0–8.0)	2.0 (0.0–8.0)	0.501	1.0 (0.0–8.0)	2.0 (0.0–8.0)	0.929
Almost 1 IADL dependent (intensive assistance or dependent), *n* (%)	91 (40.1)	383 (39.4)	0.858	108 (39.0)	366 (39.7)	0.823
Take ≥ 5 prescribed medications, *n* (%)	157 (69.2)	591 (60.9)	**0.021**	188 (67.9)	560 (60.9)	**0.035**
Quality of Life (EQoL-VAS)	75.0 (60.0–80.0)	75.0 (60.0–85.0)	**0.042**	75.0 (60.0–85.0)	75.0 (60.0–85.0)	0.812
Geriatric Depression Score (GDS > 5), *n* (%)	27 (11.9)	107 (11.0)	0.714	33 (11.9)	101 (11.0)	0.669
Visual impairments (moderate or severe), *n* (%)	128 (56.4)	531 (54.7)	0.654	145 (52.3)	514 (55.9)	0.301
Muscle mass (kg)	20.1 (16.5–26.6)	22.3 (17.4–28.3)	**0.017**	20.4 (17.2–27.8)	22.4 (17.3–28.2)	0.288
Muscle mass (kg) in men	28.2 (25.7–31.4)	28.4 (26.1–31.3)	0.580	29.2 (25.9–32.5)	28.3 (25.9–31.0)	0.248
Muscle mass (kg) in women	17.7 (15.5–19.5)	17.5 (15.7–19.7)	0.479	17.7 (15.8–19.8)	17.4 (15.7–19.6)	0.556
Handgrip strength (kg)	22.0 (18.0–30.0)	26.0 (20.0–34.5)	**< 0.001**	24.0 (18.0–30.0)	26.0 (20.0–35.0)	**< 0.001**
Osteoporosis, *n* (%)	72 (31.7)	268 (27.6)	0.215	82 (29.6)	258 (28.0)	0.607
Urinary incontinence (moderate or big problems), *n* (%)	67 (29.5)	235 (24.2)	0.097	82 (29.6)	220 (23.9)	0.055
CIRS total score	8.0 (5.0–11.0)	7.0 (5.0–11.0)	0.209	8.0 (5.0–12.0)	7.0 (4.0–10.0)	**0.001**

Data are expressed by median and interquartile range (Mann–Whitney *U* test was used for comparisons) or by number and percentage (Chi-squared test was used for comparisons)

Bold numbers indicate *p* < 0.05

Interestingly, compared to the non-fallers group, a higher percentage of women was in the group of fallers at the 12-month (59.5% vs. 50.9%, *p* = 0.02) as well as at the 24-month follow-up (61.0% vs. 49.9%, *p* = 0.001). The fallers and non-fallers also differed significantly with regard to marital status, and this difference was even more pronounced at the 24-month follow-up. With regard to medication, there was also a significant difference between fallers and non-fallers at both follow-ups, showing that the percentage of participants taking more than five prescribed medications was higher in the group of fallers at 12-month follow-up (69.2% vs. 60.9%, *p* = 0.02) and at 24-month follow-up (67.9% vs. 60.9%, *p* = 0.03). These results are supported by the CIRS scores at 24 months, indicating that fallers are reporting more comorbidities than non-fallers (8.0 vs. 7.0 *p* = 0.001). With regard to muscle mass, it was interesting to notice that there was no difference distinguishing by gender.

With regard to the main objective SPPB and falls, the SPPB (sum score and single item scores) were significantly different between fallers and non-fallers over time. Only the gait speed measure did not significantly differentiate fallers and non-fallers at 24 months (Table 2). Congruent with the SPPB Chair Rise Score, the handgrip strength also differed significantly between fallers and non-fallers (Table 1).

**Table 2:** Comparison of SPPB scores between fallers and non-fallers at 12-month and 24-month follow-up

	Fallers at follow-up 12 months (*n* = 227)	Non-fallers at follow-up 12 months (*n* = 971)	*p*	Fallers at follow-up 24 months (*n* = 277)	Non-fallers at follow-up 24 months (*n* = 921)	*p*
SPPB Balance score at baseline	4.0 (2.0–4.0)	4.0 (3.0–4.0)	< 0.001	4.0 (3.0–4.0)	4.0 (3.0–4.0)	0.001
SPPB Gait speed score at baseline	3.0 (3.0–4.0)	4.0 (3.0–4.0)	0.006	4.0 (3.0–4.0)	4.0 (3.0–4.0)	0.188
SPPB Chair stand score at baseline	3.0 (1.0–4.0)	3.0 (2.0–4.0)	0.043	3.0 (1.0–4.0)	3.0 (2.0–4.0)	0.002
SPPB total score at baseline	9.0 (7.0–11.0)	10.0 (8.0–11.0)	< 0.001	9.0 (7.0–11.0)	10.0 (8.0–11.0)	0.001

Data are expressed by median (interquartile range). Mann–Whitney *U* test was used for comparisons

At the 12-month follow-up, 121 participants reported an injurious fall, and at the 24-month follow-up, 161 participants reported an injurious fall. Hereby, 21 fractures, 48 treated and 52 untreated injuries were reported at 12-month follow-up and 30 fractures, and 52 treated and 79 untreated injuries were reported at 24-months. The SPPB neither in the total sum score nor in the single items did not differentiate if the fallers experience an injurious fall or not at both follow-ups (Table 3).

**Table 3:** SPPB comparison between persons experiencing injurious falls and non-injurious falls at 12-month and 24-month follow-up

	At least 1 injury falls at follow-up 12 months (*n* = 121)	No injury falls at follow-up 12 months (*n* = 106)	*p*	At least 1 injury falls at follow-up 24 months (*n* = 161)	No injury falls at follow-up 24 months (*n* = 116)	*p*
SPPB Balance score at baseline	4.0 (2.5–4.0)	4.0 (2.0–4.0)	0.885	4.0 (3.0–4.0)	4.0 (3.0–4.0)	0.480
SPPB Gait speed score at baseline	3.0 (3.0–4.0)	4.0 (3.0–4.0)	0.984	4.0 (3.0–4.0)	3.5 (3.0–4.0)	0.301
SPPB Chair stand score at baseline	3.0 (1.0–3.5)	3.0 (1.0–4.0)	0.661	3.0 (1.0–4.0)	3.0 (1.0–4.0)	0.897
SPPB total score at baseline	9.0 (7.0–11.0)	9.5 (7.0–11.0)	0.614	9.0 (7.0–11.0)	9.0 (7.0–11.0)	0.647

Data are expressed by median (interquartile range). Mann–Whitney *U* test was used for comparisons

Addressing possible differences for indoor and outdoor falls, we found that at 12-month 87 participants of the 227 reported a home fall and 140 reported no-home fall. With the exclusion of the SPPB balance score at 12-month follow-up, all SPPB scores (sum or single item scores) were significantly different between home fallers vs. no-home fallers. The home fallers scored 1 point less in gait speed and chair rise at both time points, and the sum score of the SPPB differed by 1 point over both follow-up time points. This reveals that participants showing less physical performance reported more falls in their home and less no-home falls (Table 4).

**Table 4:** SPPB comparison between home fallers and no-home fallers at 12-month and 24-month follow-up

	At least 1 home fall at follow-up 12 months (*n* = 87)	No home falls at follow-up 12 months (*n* = 140)	*p*	At least 1 home fall at follow-up 24 months (*n* = 104)	No home falls at follow-up 24 months (*n* = 173)	*p*
SPPB Balance score at baseline	4.0 (2.0–4.0)	4.0 (3.0–4.0)	0.519	3.0 (2.0–4.0)	4.0 (3.0–4.0)	0.003
SPPB Gait speed score at baseline	3.0 (2.0–4.0)	4.0 (3.0–4.0)	0.007	3.0 (2.0–4.0)	4.0 (3.0–4.0)	< 0.001
SPPB Chair stand score at baseline	2.0 (1.0–3.0)	3.0 (2.0–4.0)	0.004	2.0 (1.0–3.0)	3.0 (1.5–4.0)	< 0.001
SPPB total score at baseline	9.0 (5.0–10.0)	10.0 (8.0–11.0)	0.011	9.0 (6.0–10.0)	10.0 (8.0–11.0)	< 0.001

Data are expressed by median (interquartile range). Mann–Whitney *U* test was used for comparisons

### Multivariate analysis

Our multivariate analysis on the total sum of the SPPB demonstrated significant results in the crude model at 12 months and 24 months by comparing fallers to non-fallers. When we adjusted for age and sex, we only obtained significant results at 12-month follow-up. By adjusting for more variables—next to age and sex—the results did not show significant associations neither for 12-month nor for 24-month follow-up (Table 5). To identify a potential overadjustment that might have been caused a high correlation of SPPB with muscle mass and handgrip strength, models 3 and 4 were also calculated without adjusting for these two variables. However, this does not lead to relevant changes of the results [Model 3: OR 0.95 (0.90–1.01), Model 4: 0.99 (0.94–1.05)].

**Table 5:** Multivariate regression analysis between fallers and non-fallers at 12-month and 24-month follow-up

Predictors	OR (95% CI) falls at 12 months	OR (95% CI) falls at 24 months
Model 1. Compares fallers vs. non-fallers SPPB Total Score	**0.91 (0.87–0.96)**	**0.93 (0.88–0.97)**
Model 2. Adjusted for age and sex	**0.94 (0.88–0.99)**	0.96 (0.91–1.01)
Model 3. Adjusted for age, sex, marital status, number of prescribed medications, Quality of Life, muscle mass, handgrip strength	0.94 (0.87–1.02)	–
Model 4. Adjusted for age, sex, marital status, height, number of prescribed medications, handgrip strength, CIRS	–	0.99 (0.93–1.05)

Bold numbers indicate *p* < 0.05

As we did not find any results for the comparison between injurious falls and non-injurious falls, we did not perform any regression analysis.

## Discussion

Our objective of this sub-study of the SCOPE project was to investigate the predictive values of the SPPB (total sum and single domains) on falls, injurious falls, and indoor vs. outdoor falls.

The Centers for Disease Control CDC has developed an algorithm for assessing risk if falls to assist in prevention of falls [8]. One of the factors that were recommended by this algorithm is assessing functional performance which may assist in developing physical intervention programs. The SPPB was developed to measure balance gait and functional lower extremity muscle strength captures and predicts mortality, and hospitalization [22]. However, to the best of our knowledge, evidence of the predictive value of the SPPB regarding falls or injurious falls is inconclusive and rare. Most studies investigated the association of the SPPB with falls in cross-sectional study design [23, 24]. Longitudinal data with regard to the prediction of the SPPB to falls are limited and show controversial results [25]. In the systematic review by Lusardi et al. [26], the SPPB was even excluded in the term search.

Our results did show significant results of the SPPB total sum score between fallers and non-fallers over the 12-month and 24-month follow-up time in the unadjusted multivariate analysis. By adjusting for age and sex, the SPPB sum score still predicted falls over 12 months but not for 24 months. In contrast, in the fully adjusted model, we could not demonstrate that the SPPB sum score predicted fallers over a 12-month and 24-month time period.

Fall research has demonstrated that age and gender are risk factors for falls as the percentage rise with increasing age and women are at higher risk of falling [14]. Although known is that living alone is a fall risk factor and we therefore, controlled for the marital status. This was the same reason for including number of medication and the comorbidity status (CIRS). To obtain the predictive value of the SPPB alone, we further controlled for handgrip strength and muscle mass in our analysis.

Interestingly, the domain “gait speed” lost the predictive value over the 24-month time period. This result is in contrast to a just published umbrella review proposing to use gait speed in the screening process for predicting falls [9]. One explanation could be that gait speed in the SPPB is measured over a short distance (4 m) and has no run-in phase. Others may suggest that the decline in gait speed during the first year represents a situation where older people are not adapted to the reduction in their physical performance level and may not adjusted their lifestyle thus be more prone to fall. However, during the second year, they adopted a more careful lifestyle, and thus, gait speed was less likely associated to fall as a result. Research has demonstrated that differences in study protocol on gait speed produce significant different results [27].

Similar to our findings, the study by Hsieh et al. [28] found significant predictive value of the SPPB sum score in older persons. Their population had a younger mean age (73.02 years). In congruence with our findings, the study by Hsieh also demonstrated by adjusting for confounding variable in multivariate analyses, and the SPPB lost the predictive value [28]. They also adjusted in their multivariate analysis for age, sex, and number of medication comparable to our adjustments. Similar findings were obtained by Li et al. in a Chinese cohort with a 5 year follow-up time period [29].

In contrast to our study, the prospective study by Pettersson et al. [30] did not find a predictive value of the SPPB over a 12-month follow-up time period. Her study included only 202 older community-dwelling persons from a previous study with a mean age of 79 years. Participants of this study used a daily calendar to report falls and in case of a reported fall had a structured interview. The participants of the Pettersson’s study are comparable with our participants with regard to age, cognition (MMSE), and a baseline SPPB sum score of 10. In contrast to our study, 71% of the participants were women. Nevertheless, nearly half of the included quite healthy participants reported a fall during the 12-month follow-up period which is different to our study.

A recent study by Welch et al. [31] that only lower scores in the SPPB (sum score of 3–6) had a three times higher risk for falls then participants with SPPB scores 10–12. Over the 4-year time period, even after adjusting for other fall-risk factors, a low SPPB sum score and low gait time in the SPPB predicted higher fall risk. In our study, the lowest SPPB sum score was 7 which could explain the lost significant in the adjusted model.

Kerber et al. [32] investigated the prediction of experiencing ≥ 2 falls with trajectories of the SPPB and found that even including a trajectory over time of the physical performance (measured with the SPPB) did not improve meaningfully the baseline prediction on falls. One major difference to our study was that they defined a faller with ≥ 2 falls, and we defined a faller with already one reported fall event, and that they included a trajectory of the SPPB scores over time.

With regard to the question, if the SPPB could predict injurious falls in community-dwelling older persons, we did not find significant results in our cohort. Another longitudinal study investigated the predictive value of the SPPB on injurious falls [33]. Falls were obtained with a daily calendar over a 4-year period. Injurious falls were defined resulting in fractures, sprains, dislocation, pulled or torn muscles, or seeking medical attention. The study by Ward et al. [33] showed over a median follow-up time of 2.4 years that 29% of their participants experienced one or more injurious falls. The included participants were similar to ours with a mean age of 78.1 year and 64.1% of women. They found no significant prediction of the SPPB total score but by looking at the single domains the participants with the lowest score on the chair rise domain of the SPPB had a greater hazard ration of 16% then all other chair rise categories [33]. No significant predictive results were also reported for the other two domains of the SPPB (gait speed and balance).

Our third objective was to investigate the predictive value of the SPPB and its three sub-scores on possible differences between home and no-home falls. Here, we found again significant differences on the SPPB total sum score as well as the strength and gait speed sub-score over the 12-month and 24-month period. This time, the balance sub-score differentiated between indoor and outdoor falls only over the 12 months and did not over 24 months. A study by Kelsey et al. [25] investigated also in differences between indoor and outdoor falls in community-dwelling older persons. They followed their 70 years and older participants over a mean time of 21.7 months. They found that ADL impairment were more predictive for indoor falls than outdoor falls (HR 2.57 vs. 0.27). This supports our results that outdoor falls seem to be more prevalent in the higher active older persons what also had been reported in an earlier study [34].

What is the clinical implication of our study? The SPPB is a well-known and validated tool including the most important fall-risk factors (strength, balance, and gait speed). In addition, the SPPB is now even approved by the European Medical Agency (EMA) for categorization of the frailty status, thus will be gaining even more importance (35). In 2017, this reflecting paper stated that “The SPPB is the preferred option for routine use in clinical trials to characterize baseline physical frailty, as it has the best prognostic value of disability and mortality”.

What does all the data of the predictive value of the SPPB tell us? The definition of falls used in all the cited studies were quite similar which shows the impact of the suggested definition either by the WHO or the former Prevention of Falls Network Europe (ProFaNE) group [1, 35]. As our results did not show the predictive value of the SPPB in the adjusted multivariate analysis, it supports the just published algorithm including more than just the physical function domain e.g., concerns about falling and falls history [3]. This is supported by a recently published paper.

With regard to injurious falls, the picture is not so clear, as different definitions were used in the cited studies. Future studies should probably use the categorization by Schwenk et al. [36] for reliable comparison of studies. Our data did also not fulfill this categorization nevertheless we obtained different levels of injuries. As fall-related injuries are responsible for mortality, disability and loss of independence [37], more research is needed as in most fall prevention statements the differentiation between falls only, and injurious falls is not well stated [1, 3, 14], but should be made in future studies. This of course needs to be addressed in the screening process on fall-risk and relevant assessment tools with regard psychometric properties of the proposed physical assessment tools (e.g., gait speed, or TUG or SPPB) by the official guidelines.

Studies investigating probable differences between home and outdoor falls are just coming into focus [25]. The interaction between the environment as well as the functional status on falls or injurious falls have been acknowledged in many fall prevention guidelines [1] but are much less investigated with regard to falls at home or outside. The assumption that indoor falls occur mostly in frail older persons and outdoor falls are more common in high active older persons need to be investigated much more as it would mean different intervention approaches. For frail older persons, a multi-component exercise program might be much more appropriate than for high-functioning outdoor fallers. The latter persons might need much more an intervention focusing on behavioral aspects of fall prevention.

Of course, our study has strength and limitations. Our study could use the longitudinal data of the SCOPE project addressing community-dwelling older persons from several European countries and Israel. The prospective time periods of 12 months and 24 months are important markers in fall research in this clinically relevant population. A further strength of this study was the analysis of falls occurring at home vs. out of home, adding additional information to the current knowledge. Another important strength of the study is the nearly equal distribution between sexes. Furthermore, by including the SPPB as being predictive on falls, we extend the current predictive value of the SPPB, being evaluated mostly on mortality, nursing home admission, or hospitalization.

Even though, we have to state also some limitations of our study. First of all, this is a sub-study of the SCOPE project not designed for fall detection. We only included the participants with no falls history at baseline but reporting a fall event during the follow-up time period. Furthermore, falls, injurious falls, as well as circumstances of falls were obtained self-reported which probably pose a recall bias in the obtained number and information on falls. Especially, falls were recorded for a period of 12 months, respectively, without a fall diary or regular interviews in the meantime which likely leads to an underreporting of falls. Additionally, particularly those participants could be lost to follow-up that had injurious falls or even fall-related fatality. On recruitment strategy of SCOPE was the inclusion of persons visiting outpatient clinics. This could have led to selection bias, because the initial assessment could be altered by an illness or other health issue.

## Conclusion

Our results did show the predictive value of the SPPB over 12 months and 24 months in univariate analysis, but these results could not be maintained in the multivariate analysis when we adjusted for other variables. Our longitudinal study provides more insight in the predictive value of the SPPB on falls as well as on injurious falls. We also could support the existing knowledge that there is a difference between indoor and outdoor falls based on the physical functional level.
